# Active SAmpling Protocol (ASAP) to Optimize Individual Neurocognitive Hypothesis Testing: A BCI-Inspired Dynamic Experimental Design

**DOI:** 10.3389/fnhum.2016.00347

**Published:** 2016-07-07

**Authors:** Gaëtan Sanchez, Françoise Lecaignard, Anatole Otman, Emmanuel Maby, Jérémie Mattout

**Affiliations:** ^1^Center for Cognitive Neuroscience, University of SalzburgSalzburg, Austria; ^2^Lyon Neuroscience Research Center, Brain Dynamics and Cognition Team, Institut National de la Santé et de la Recherche Médicale, U1028, Centre National de Recherche Scientifique, UMR5292, Université Claude Bernard Lyon 1Lyon, France; ^3^CERMEP Imaging CenterLyon, France

**Keywords:** adaptive sampling protocol, brain-computer interface, adaptive design optimization, generative models, sequential hypothesis testing, dynamic causal modeling, bayesian model comparison, bayesian inference

## Abstract

The relatively young field of Brain-Computer Interfaces has promoted the use of electrophysiology and neuroimaging in real-time. In the meantime, cognitive neuroscience studies, which make extensive use of functional exploration techniques, have evolved toward model-based experiments and fine hypothesis testing protocols. Although these two developments are mostly unrelated, we argue that, brought together, they may trigger an important shift in the way experimental paradigms are being designed, which should prove fruitful to both endeavors. This change simply consists in using real-time neuroimaging in order to optimize advanced neurocognitive hypothesis testing. We refer to this new approach as the instantiation of an Active SAmpling Protocol (ASAP). As opposed to classical (static) experimental protocols, ASAP implements online model comparison, enabling the optimization of design parameters (e.g., stimuli) during the course of data acquisition. This follows the well-known principle of sequential hypothesis testing. What is radically new, however, is our ability to perform online processing of the huge amount of complex data that brain imaging techniques provide. This is all the more relevant at a time when physiological and psychological processes are beginning to be approached using more realistic, generative models which may be difficult to tease apart empirically. Based upon Bayesian inference, ASAP proposes a generic and principled way to optimize experimental design adaptively. In this perspective paper, we summarize the main steps in ASAP. Using synthetic data we illustrate its superiority in selecting the right perceptual model compared to a classical design. Finally, we briefly discuss its future potential for basic and clinical neuroscience as well as some remaining challenges.

## Introduction

A major goal in any empirical science is alternative hypothesis testing^1^. Hypothesis testing is also at the heart of individual evaluations such as clinical diagnoses or school exams. Applied statistics provide the methods to design such tests, to compare hypotheses and to eventually estimate parameters of interest with the aim of making the best decisions.

This process entails the following main steps:

1. Stating the alternative hypothesis (or hypotheses);
2. Designing the experiment;
3. Acquiring the data;
4. Analyzing the data;
5. Concluding.

In neuroscience, step (1) pertains to the question of interest regarding brain functions, mental processes and neurophysiological phenomena. Note that hypotheses in step (1) are sometimes and deliberately not precisely defined, following what is often referred to as an exploratory approach. However, the more refined the initial hypotheses, the more straightforward the design of the experiment.

Step (2) consists of setting all the acquisition parameters. This implies defining the measures to be performed (behavioral, electrophysiological or neuroimaging…), the population from which to sample (healthy individuals or patients, young or elderly people, females or males…), the sample size, the stimulations to be used, the task that will be assigned to the participants, the instructions that will be given, as well as the timing and length of the experiment.

Importantly, step (2) becomes increasingly crucial as experimental cost increases, which is the case when scanning time is expensive, when the sample size needs to be high, or when it is difficult to recruit from the targeted population (e.g., patients with rare pathologies). Therefore, step (2) often includes a piloting phase which consists of testing a couple of participants in order to confirm or adjust the experimental design before moving to step (3).

After data acquisition, step (4) typically consists of preprocessing and model fitting. Finally, step (5) concludes from formal model comparison and parameter estimation based on the best model or best model family, if any.

At the end of this serial process, the worst case scenario occurs when the experiment concludes in favor of an incorrect hypothesis. Another undesirable scenario is when the whole experiment is inconclusive, as when there is not enough evidence to favor one hypothesis over the others.

One of those two scenarios can arise from at least two possible and mutually non-exclusive explanations, namely that:

- The design was not optimal for disentangling the competing hypotheses;
- The amount of data acquired was insufficient (e.g., more trials from a given participant, or more participants from a given population were necessary).

Crucially, in cognitive neuroscience, two factors may contribute and even interact to make this pitfall particularly acute. First, both the within- and between- subject variability are potentially large and difficult to estimate. Second, as our field progresses and the hypotheses to be tested become more subtle and complex, it can be very difficult to foresee how much their respective predictions do or do not differ. In other words, in the absence of any prior information or measure, the noisy nature and incompleteness of the data together with model complexity, can make the task of optimizing the experimental design particularly difficult.

These general considerations highlight the main decision criterion that governs experimental design, namely optimizing our chances of concluding, or in other words, minimizing the risk of hypothesis testing errors.

Interestingly, this criterion can now be optimized during an experiment, using the principle of sequential hypothesis testing pioneered by Wald (1947) and its modern instantiations such as the one proposed by Myung and Pitt in the Cognitive Science field (Myung et al., 2013).

This simple idea could trigger a methodological change in cognitive neuroscience for at least two reasons:

- It is now possible to implement such a sequential optimization process, thanks to our ever-growing ability to process large neuroimaging and electrophysiological datasets online. This is promoted by the development of real-time brain imaging and the field of brain-computer interfaces.
- Bayesian inference and Bayesian decision theory provide a generic and flexible framework which enables online model comparison as well as online design optimization, for a large variety of complex (dynamical, non-linear) hypotheses.

In what follows, we briefly review these two aspects which are the building blocks of ASAP. We then illustrate the potential of ASAP on a toy example that concerns disentangling alternative models of perception when observing a sequence of stimulations. Finally, we briefly discuss some challenges and perspectives for the future development of ASAP.

## Brain-computer interfaces and real-time neuroimaging

The term Brain-Computer Interface (BCI) was coined by Jacques Vidal, a visionary engineer (Vidal, 1973). However, the field only emerged two or three decades after it was named, when computer power and brain acquisition techniques enabled the real time processing of brain signals. Since then, and although BCI is still in its infancy (an international society has only just been created), the community has grown tremendously, and applications have widened accordingly (e.g., Moxon and Foffani, 2015), and there is now a demand for the development of advanced wearable acquisition devices (Mullen et al., 2015). Today, BCI requires not only engineers but also neuroscientists and clinicians to tackle its multidisciplinary challenges.

Less than a year ago, a European consortium of BCI experts drew a roadmap for the field (Brunner et al., 2015). They defined BCI as a system which (i) converts brain signals into an estimated brain state or command for a machine, (ii) operates online, and (iii) provides (explicit or implicit) feedback to the user. This consortium also listed the main categories of applications that BCI is targeting. These includes “*replacing*” a lost function (e.g., with neuroprosthetics), “*restoring*” a lost function (e.g., through brain-controlled functional-electric-stimulation), “*improving*” rehabilitation protocols by including direct brain measures in the loop (e.g., for biofeedback-based rehabilitation after stroke), “*enhancing*” an interaction, for instance a learning path, by monitoring states such as vigilance and mental work-load. Finally, they acknowledge a last but not least scenario which is the usefulness of BCI for research.

Since the aim of BCI is to decode brain activity, it is tightly bound to research studies on the neural correlates of various mental states. Exploring how BCI decoding performance evolves with the nature, the number, the size, and the variety of the recorded neuronal populations thus provides important insights into how the brain encodes information (Nicolelis and Lebedev, 2009).

More importantly, at least for us, is that the BCI loop instantiates a dyadic system in which the brain not only adapts to the ongoing interaction, but the machine also adjusts its behavior online (Mattout, 2012). This adjustment should be driven by the objective of the BCI and based on the sequence of brain states that can be inferred online from brain activity. Whereas typical BCIs have the clear objective to optimize the interaction for a given patient (e.g., autonomy of movement, communication…), ASAP offers a generic and principled way to optimize another objective: neurocognitive hypothesis testing.

ASAP relies on Bayesian inference for model fitting and model comparison. This corresponds to the perceptual and learning part of the Active Sampling process. The Bayesian framework and the type of models ASAP can deal with are summarized in the next section.

Adjusting the experimental design online relies on a Bayesian decision-theoretic criterion which prescribes the maximization of design efficiency or, equivalently, the minimization of the error risk in model comparison. This step corresponds to the action part of the Active Sampling process. It is described in the next section.

## Computational neuroscience and model-based neuroimaging

Functional neuroimaging has evolved toward the use of more and more advanced models to refine the types of neurocognitive hypotheses that can be tested with either PET, fMRI, EEG, or MEG (Friston, 2009). These models are generative models of how brain responses are shaped and modulated by incoming sensory inputs and contextual factors. In their most generic form, they are referred to as dynamic causal models (DCM; Friston et al., 2003; Stephan et al., 2007) and write

(1) $$\begin{array}{l} ẋ=f\left(x,\theta ,u\right) \end{array}$$

$$\begin{array}{l} y=g\left(x,\varphi ,u\right)+\varepsilon \end{array}$$

where unobserved or hidden state variables *x* evolve in time as prescribed by function *f*, depending on current state *x*, unknown parameters θ and external inputs or contextual factors *u*. States *x* map onto observed data *y* through function *g* which depends on unknown parameters φ. Data *y* are usually corrupted by some additive noise ε.

Let us consider the modeling of EEG or MEG signals. Then *x* typically describes some neuronal states in a network of interacting brain regions. θ typically refers to the connection strength between brain regions, while *u* may indicate the nature of the stimulus. *y* corresponds to measured signals in space and time. A few examples of DCM include characterizing differences in evoked (David et al., 2006), induced (Chen et al., 2008), or steady-state responses (Moran et al., 2009) in terms of modulations of effective connectivity in a cortical network. For instance, it has been used to explain mismatch responses to deviant auditory stimuli and has helped distinguish between minimally conscious patients and patients in a vegetative state (Boly et al., 2011).

Interestingly, the same kind of models can be used to represent mental processes such as learning and decision making (Mathys et al., 2014). In these cases, the evolution function *f* embodies the learning process which updates the hidden cognitive variable *x* (e.g., the probability of getting a reward when faced with a two alternative choice *u*), depending on parameters θ (e.g., the confidence in predicting the reward associated with each alternative). Then state *x* predicts the observable outcome *y* (e.g., the participant's choice). This kind of model predicts behavior but can also be used in model-based neuroimaging to distinguish between learning styles in certain contexts (Behrens et al., 2008).

These models mentioned above pertain to how an experimenter represents the physiological or psychological processes at play in the subject's brain. Importantly, one can assume that the subject entertains the same kind of models to represent the generative process a computer program is implementing, so that he can predict future sensory inputs and optimize his behavior (Daunizeau et al., 2010). This view is in line with the so-called Bayesian brain hypothesis and the Free-energy principle (Friston et al., 2011), whereby, in a common framework, perceptual learning and inference are necessary to induce prior expectations about the observable data. Furthermore, action is engaged to resample the world to fulfill expectations. This places perception and action in intimate relation and accounts for both with the same principle.

In contrast with the above examples, the meanings of variables *u* and *y* are swapped here. Indeed, the subject now entertains a model of its environment whose outcome is the displayed stimulation *y*, which may or may not depend on some earlier cue *u* (possibly incorporating the subject's earlier actions).

In the above example of monitoring probability *x* of getting a reward by selecting a particular action, the way the subject updates *x* over trials relies itself on inverting a DCM predicting how the computer is generating those rewards. This is referred to as a meta-Bayesian approach in the sense that a probabilistic model of how the subject is perceiving the task is encapsulated in a probabilistic model of how the measured (physiological or psychological) data are generated from the hidden perceptual variables (Daunizeau et al., 2010). In this sense, the meta-Bayesian approach incorporates all the previously-mentioned model-based approaches that are used in neuroimaging nowadays and which are at the heart of the new field of computational psychiatry in particular (Adams et al., 2016).

## Principles of Active SAmpling Protocol (ASAP)

DCMs can be inverted and compared using Bayesian techniques. Moreover, variational techniques and the Laplace assumption (Friston et al., 2007) provide robust approximate inference on models and model parameters which can be performed online (Sanchez et al., 2014). Here are the main computational aspects of this online inference process.

Consider *K* alternative models {*M*_*k*_}_*k*∈[1, *K*]_. At each trial *t*, the posterior probability of each model will be updated based on prior knowledge and new observation *y*_*t*_ following Bayes rule

(2) $$\begin{array}{l} p\left(M_{k}|y_{t},u_{t}\right)\propto p\left(y_{t}|M_{k},u_{t}\right)p_{t}\left(M_{k}\right) \end{array}$$

under the constraint

(3) $$\begin{array}{l} \underset{k\in \left[1,K\right]}{\sum }p\left(M_{k}|y_{t},u_{t}\right)=1 \end{array}$$

At the beginning of the experiment, prior to any observation, all models are usually considered as equally likely

(4) $$\begin{array}{l} \forall \;k\in \left[1,K\right],\;p_{0}\left(M_{k}\right)=\;1∕K \end{array}$$

then Bayesian learning relies on updating the prior over models with the latest posterior

(5) $$\begin{array}{l} \forall \;k\in \left[1,K\right],\;p_{t}\left(M_{k}\right)=\;p\left(M_{k}|y_{t-1},u_{t-1}\right) \end{array}$$

Finally, model log-evidence log *p* (*y*_*t*_ | *M*_*k*_, *u*_*t*_) is approximated by the variational free energy *F*_*k, t*_. In variational Bayes, the free energy is maximized iteratively. At convergence, it can be used for model comparison since it expresses a trade-off between the quality of fit and model complexity (Penny, 2012).

Importantly for ASAP, the free energy of each model and hence model posteriors depend on design variable *u*_*t*_. This means that at each trial, after having updated our belief from past observations, one could choose the next design parameter *u*_*t*_ so as to optimize some criterion over model-free energies. Since we are interested in optimizing model comparison, the most natural criterion is the minimization of the risk of a model selection error. For general DCMs, as described above, it has been shown that this risk *Q*_*e*_ can be approximately minimized with respect to design variable *u*_*t*_ by maximizing the Jensen-Shannon divergence, also referred to as the design efficiency (Daunizeau et al., 2011)

(6) $$\begin{array}{l} D_{JS}\left(u_{t}\right)=H\left(\underset{k\in \left[1,K\right]}{\sum }p\left(y_{t}|M_{k},u_{t}\right)p_{t}\left(M_{k}\right)\right)\\ -\underset{k\in \left[1,K\right]}{\sum }p_{t}\left(M_{k}\right)H\left(p\left(y_{t}|M_{k},u_{t}\right)\right) \end{array}$$

where *H* indicates Shannon's entropy.

**Figure 1:**
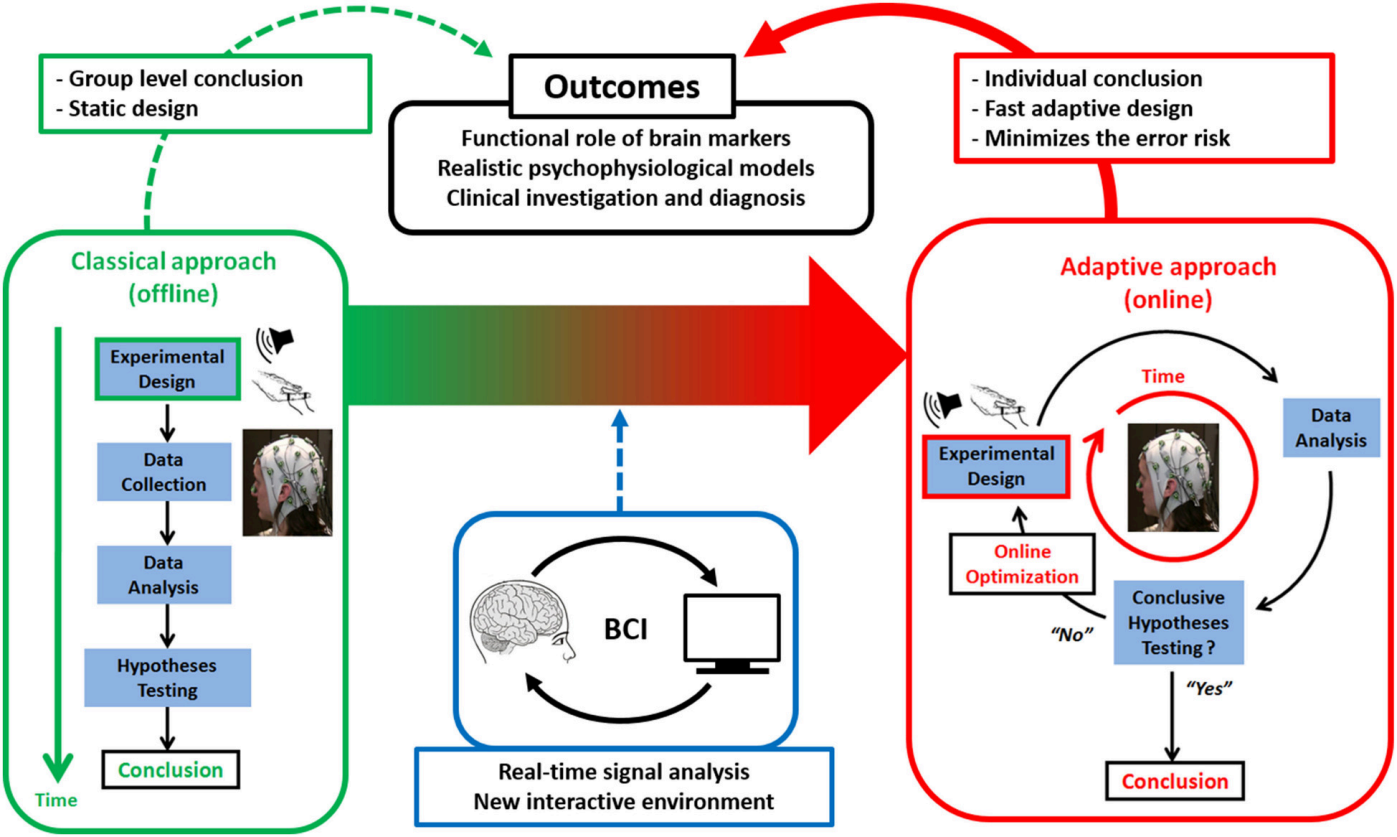
**Active SAmpling Protocol (ASAP)**. General principles of ASAP (right red panel) vs. classical (left green panel) designs as enabled by applying principles from Brain-Computer Interfaces (BCI), namely processing human electrophysiological or neuroimaging data online to optimize basic or clinical neurocognitive hypothesis testing.

At trial *t*, design optimization consists in selecting the design variable *u*_*t*_ that maximizes the above Jensen-Shannon entropy. At each trial, ASAP thus alternates between optimizing the free energy for each model and computing the design efficiency for each possible design value *u*_*t*_. This is the principle of Active Sampling or Active Inference (Friston et al., 2011), whereby action is guided by online inference and vice versa. It is depicted in Figure 1, in contrast to classical (offline) experimental design.

We have previously shown in earlier simulations that ASAP can yield more accurate and faster experiments (Sanchez et al., 2014). Using a new toy example, we here illustrate the type of adaptive design ASAP may come up with in order to disentangle alternative models of perception.

## Synthetic example: disentangling between alternative perceptual models

We consider a virtual task where subjects are passively observing a sequence of stimulations which can only take two values: a frequent and a rare one. What may vary in the design (variable *u*) is the number of frequent stimuli in between two rare stimuli. This theoretical situation is reminiscent of oddball paradigms where a component known as the mismatch negativity (MMN) can be measured with EEG and has been linked to prediction error in perceptual learning (Lecaignard et al., 2015). In our example, we assume we capture a noisy measure of prediction error (the data *y*) after each rare stimulus has been perceived.

We consider 3 alternative models. For each model, the following equations give the outcome of the predicted response, namely the proxy *y*_*t*_ for the evoked response at trial *t*:

The “Null” model (NM) simply predicts noisy data with no dependency on the sequence of stimuli.

(1) $$\begin{array}{l} y_{t}=\;C_{0}+\;\varepsilon \end{array}$$

$$\begin{array}{l} \varepsilon \;\sim \;N\left(0,\sigma \right) \end{array}$$

The “Change Detection” model (ChangeD) produces a binary output reflecting the detection of a change in the number of preceding frequent stimuli, from one rare stimulus to the next.

(2) $$\begin{array}{l} y_{t}=\{\begin{array}{c} C_{0}+\;\varepsilon ,if\;u_{t}=\;u_{t-1}\\ C_{1}+\;C_{0}+\;\varepsilon ,if\;u_{t}\neq \;u_{t-1} \end{array} \end{array}$$

The “Learning” model (LM) updates a prediction of the exact number of preceding frequent stimuli. Similar models have been explored to explain trial-to-trial changes in EEG evoked responses in oddball paradigms (Ostwald et al., 2012).

(3) $$\begin{array}{l} y_{t}=\;BS\left(p\left(x_{t}|u_{t}\right),\;p\left(x_{t-1}\right)\right)*\;C_{1}+C_{0}+\varepsilon \\ \text{ where }\{\begin{array}{c} x\;\sim \;Gamma\;\left(G,D\right)\\ G^{t}=\text{ exp}\left(-\frac{1}{\tau }\right)*\;G^{t-1}+\;u_{t}\\ D^{t}=\text{ exp}\left(-\frac{1}{\tau }\right)*\;D^{t-1}+\;1 \end{array} \end{array}$$

Where *BS* indicates the Bayesian surprise or Kullback-Leibler divergence between the prior and posterior distributions over state *x*_t_ which is the predicted number of preceding frequent stimuli. *x*_*t*_ is updated following Bayes rule but assuming a forgetting mechanism with single parameter τ as in (Ostwald et al., 2012).

In our simulations, parameters *C*_0_ and *C*_1_ were set to 0.7 and 0.5, respectively. τ was set to 5 and the noise precision or inverse variance was fixed at 10.

We compare ASAP with a classical roving-type design which introduces volatility in stimulus presentation and has been already used with success to reveal implicit perceptual learning processes (Ostwald et al., 2012). With each design, we simulated 50 different subjects under (true) model LM or ChangeD, in 200-trial experiments. These simulations were performed with the VBA toolbox (Daunizeau et al., 2014).

**Figure 2:**
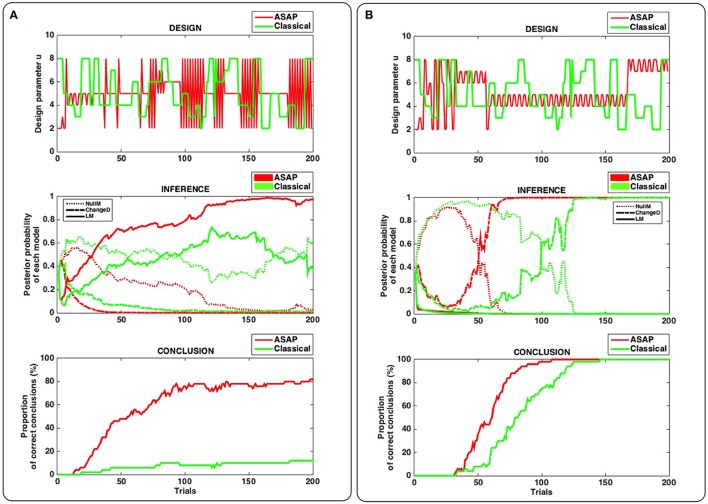
**Results of synthetic experiments comparing ASAP with a classical (static) design (see main text for details)**. **(A)** True model used for simulation = “Learning” model (LM). **(B)** True model used for simulation = “Change Detection” model (ChangeD). Top panel: single example of a stimulation sequence produced by ASAP (red) and by a roving-type classical design (green). Middle panel: dynamics of model posterior probabilities over trials, for the example shown in the top panel. For each experimental design, the plain, dashed and dotted lines correspond to models LM, ChangeD, and NullM, respectively. Bottom panel: Proportion of correct conclusions over the 50 simulated experiments for each design.

Figure 2 illustrates our main results. Bottom panels in Figures 2A,B enable us to compare ASAP with a classical (static) design, on average.

Interestingly, when LM is the true model (Panel A), that is a fairly complex dynamical model whose behavior will depend upon the history of stimulations, ASAP proves able to conclude much more often than the classical design after 200 trials (in ~80% and less than 20% of the simulated experiments, respectively). In contrast, when ChangeD is the true model (Figure 2B), that is the most common model used to study deviance responses, the classical design is sufficient to conclude. However, ASAP proves significantly faster, enabling to shorten the experiment by more than a quarter.

These results emphasize two important strengths of ASAP. On the one hand, even though appropriate designs could be defined in a static fashion, prior to any data acquisition, like when having to identify a fairly simple (static) model, ASAP proves faster in concluding. On the other hand, when no obvious design can be defined in advance, especially in the case of complex dynamical true models, ASAP proves able to reach conclusion by coming up with an appropriate and more complex design, while the classical design simply fails to conclude.

Although somewhat anecdotal, the two detailed simulation examples (Figures 2A,B, top and middle) do highlight these two aspects in a useful way. Remember that the same roving classical design is used in both cases (see green curves on top panels A and B).

Interestingly, when ChangeD is the true model (the model for which a roving paradigm is well-suited, Figure 2B), the correct conclusion is eventually reached with the classical design, after enough data have been acquired in order to reject the null model (NullM). Model LM is appropriately discarded due to its higher complexity and its inadequacy compared to ChangeD in accounting for trial wise observations. ASAP exhibits the same kind of dynamics in model comparison but is much faster in reaching the correct conclusion. On top Figure 2B (ASAP's design, red line), it can be seen that a first and short phase consists of a highly volatile design, followed by a longer phase made of a fairly stable one. The first phase is efficient in discarding LM while the following phase is efficient in disentangling between ChangeD and NullM.

Now when LM is the true model (Figure 2A), the classical design succeeds in discarding ChangeD but fails in disentangling LM from NullM. This is because in presence of noisy data, this design is not volatile enough to conclude in favor of a complex model like LM. In contrast, ASAP is successful in picking up LM as the right model. Again, ASAP seems to follow two phases in this example. In the first short phase, ASAP uses a fairly stable design that is efficient in discarding ChangeD, while the second and long phase exhibits a complex volatile pattern which enables to conclude in favor of the true complex dynamical model (LM).

## Conclusion

Active SAmpling Protocol (ASAP) builds on advances in real-time neuroimaging, brought about by Brain-Computer Interfaces. ASAP aims to optimize neurocognitive hypothesis testing. This optimization rests on the principle of sequential hypothesis testing, namely adaptive design optimization (Myung et al., 2013). In the Bayesian framework, this optimization can be generalized to one of the most generic forms of generative models, namely dynamic causal models (Daunizeau et al., 2011). On simulations, we showed earlier (Sanchez et al., 2014), and confirmed here, that ASAP can conclude better and faster than classical designs.

Here we highlight the importance of ASAP for adaptively optimizing hypothesis testing. The same principle, however, applies to the optimization of parameter estimation and ASAP can also be extended to the comparison of families of models (Daunizeau et al., 2011). Beyond the optimization of experiments at the level of each individual, it can also be used to better design group-based clinical trials (Wathen and Thall, 2008).

The ideas behind ASAP have recently attracted attention in various fields. For instance adaptive designs were used to optimize parameter estimation of psychophysical functions (Kujala and Lukka, 2006) or single neuron responses (Lewi et al., 2009). In the latter, the authors also use an information theoretic measure to select the most appropriate stimuli. Although ASAP could also optimize the estimation of model parameters, we here emphasize another and broader objective, namely hypothesis testing, which calls for a different optimization criterion.

In the field of functional neuroimaging (fMRI), a few studies have also used dynamical design approaches. Only to mention the most related ones, those studies aimed at efficiently exploring a large stimulus space in the aim of identifying the stimulus subspace that best maps onto some given brain state (e.g., the activity pattern in a given brain region) (Cusack et al., 2012; Lorenz et al., 2016). Importantly, while these approaches are informative in revealing the existence of a particular mapping between stimulus space and some targeted brain responses, they do not consider explicit hypothesis about the mapping between the two. In contrast, ASAP provides a formal way of optimally comparing alternative generative models, relating incoming inputs or instructions with observations (neuronal, behavioral or both).

We showed that ASAP can be applied in a generic fashion to a wide range of mathematically-defined hypotheses. We advocate that its application may trigger a paradigm shift in cognitive neuroscience by promoting hypothesis-driven and individually-tailored experiments that will be optimized both in terms of their conclusions and durations. Furthermore, because they are based on explicit mathematical models, these experiments should be easier to reproduce. They may also enable inter-subject variability to be formally addressed. Clinically, they may prove particularly useful in developing quantitative and specific diagnostic approaches, which is a highly relevant objective in modern psychiatry. Finally, optimizing the experiment's length and the total number of subjects required for group studies may prove economically advantageous.

However, some important challenges need to be addressed before ASAP could be used routinely. One of the most important is the need to deal with artifacts online. Clearly, this is not a trivial task, as one has to correct or reject data based on only a few observations and under tight time pressure. Our current simulations consider noisy but artifact-free data. Empirically, it is important to compare ASAP to the traditional offline approach on real (artifacted) data. Depending on the task and features of interest, the predicted advantage to ASAP might be overestimated in such simulations. Several arguments suggest that its advantage should remain significant, however, even though artifact correction or rejection might not be as efficient as in off-line data processing. First, this challenge is being shared with all BCI applications and a lot of progress has already been made in this direction (see for example Barachant et al., 2013). Second, since ASAP is an hypothesis-driven approach, only part of the data features are processed online which helps to reduce the nuisance of artifacts, simply because only part of the data in space, time, and frequency will be analyzed. Finally, even when some physiological responses have to be discarded ASAP can still update the subject's belief given the presented stimulus, the hypothesized model, and the possible behavioral response. Of course, the cost of this will be a longer experiment.

Another aspect is the computational burden. Depending on the task, its timing, the number and complexity of the alternative hypotheses, and the number of alternative design values, ASAP might or might not be able to make the required computation fast enough. This will have to be explored empirically and optimized as much as possible.

Finally, important extensions include applying the principle of Adaptive Design Optimization not only to single subjects but also at the population level (Kim et al., 2014), as well as questions like what horizon in time should be considered for online design optimization. Provided that those crucial points can be addressed, we envisage that ASAP may prove particularly useful for diagnosis, especially as the field progresses toward the identification of functional markers of psychiatric and neurological diseases.

## Author contributions

GS and JM conceived the approach. GS, FL, AO, EM, and JM designed and implemented the simulations. GS and JM analyzed the results and wrote the paper. The current draft was approved by all authors.

### Conflict of interest statement

The authors declare that the research was conducted in the absence of any commercial or financial relationships that could be construed as a potential conflict of interest.

## Footnotes

^1^As is common in the field of neuroimaging, we use the terms hypothesis testing and model comparison interchangeably throughout the paper. In the context of ASAP, however, it is important to note that models are formulated mathematically and compared in a Bayesian framework.
